# Man/Cel5B, a Bifunctional Enzyme Having the Highest Mannanase Activity in the Hyperthermic Environment

**DOI:** 10.3389/fbioe.2021.637649

**Published:** 2021-03-16

**Authors:** Beenish Sadaqat, Chong Sha, Parveen Fatemeh Rupani, Hongcheng Wang, Wanbing Zuo, Weilan Shao

**Affiliations:** Biofuels Institute, School of the Environment and Safety Engineering, Jiangsu University, Zhenjiang, China

**Keywords:** hyperthermophilic β-mannanase, bifunctional mannanase, highest mannanase activity, mannooligosaccharides, *Thermotoga maritima*

## Abstract

*Thermotoga maritima* (*Tma*) contains genes encoding various hyperthermophilic enzymes with great potential for industrial applications. The gene TM1752 in *Tma* genome has been annotated as cellulase gene encoding protein Cel5B. In this work, the gene TM1752 was cloned and expressed in *Escherichia coli*, and the recombinant enzyme was purified and characterized. Interestingly, the purified enzyme exhibited specific activities of 416 and 215 U/mg on substrates galactomannan and carboxy methyl cellulose, which is the highest among thermophilic mannanases. However, the putative enzyme did not show sequence homology with any of the previously reported mannanases; therefore, the enzyme Cel5B was identified as bifunctional mannanase and cellulase and renamed as Man/Cel5B. Man/Cel5B exhibited maximum activity at 85°C and pH 5.5. This enzyme retained more than 50% activity after 5 h of incubation at 85°C, and retained up to 80% activity after incubated for 1 h at pH 5–8. The K_*m*_ and V_*max*_ of Man/Cel5B were observed to be 4.5 mg/mL galactomannan and 769 U/mg, respectively. Thin layer chromatography depicted that locust bean gum could be efficiently degraded to mannobiose, mannotriose, and mannooligosaccharides by Man/Cel5B. These characteristics suggest that Man/Cel5B has attractive applications for future food, feed, and biofuel industries.

## Introduction

Mannan is a complex biopolymer, composed of mannose residues that are linked together by β-1,4 linkages named as pure mannan, or the polysaccharide may be a mixture of β-1,4-D-mannose and β-1,4-D-glucose units termed as glucomannan. These mannan and glucomannan polysaccharides may also be linked to α-1,6-linked galactose residues and the corresponding polysaccharides are called as galactomannan and galactoglucomannan, respectively. Generally, 1,4-β-D-Mannan mannanohydrolase or endo-1,4-β-mannosidase (EC 3.2.1.78) is called as β-mannanase, which causes random cleavage of β-mannosidic linkages in mannan backbone (Zhu et al., 2020). Depending on sequence similarity, mannanases from various sources, such as bacteria, fungi, and plants, have been categorized within glycoside hydrolase families 5, 26, 113, and 134 within carbohydrate-active database (Ghosh et al., 2013).

β-mannanase plays an essential role in the degradation of lignocellulosic biomass as β-mannan is the principal component of hemicellulose in soft woods. β-mannanase has also been employed for the degradation of mannan in the cell wall of palm kernel cake for ethanol or biobutanol production (Cerveró et al., 2010; Shukor et al., 2016). In addition, it can degrade mannan-rich agricultural crop residues, such as locust bean gum (LBG) and guar gum into mannooligosaccharides (MOS) which have numerous health benefits (Katrolia et al., 2013). β-mannanase is extensively used in the paper/pulp, food, and feed industries, particularly in poultry feeds and prebiotic food supplements to decrease the immunogenic effect of mannan polymers, clarify fruit juices, and extract of oil from copra and detergent (Kaira et al., 2016; Singh et al., 2019). In these industries, thermostable enzymes are preferentially used (Dhawan et al., 2016; Li and Nie, 2016) due to their robustness and enhanced rate of hydrolysis (Katsimpouras et al., 2016; Niu et al., 2017). Moreover, thermozymes offer unique features such as high pH, solvent concentrations (Liu et al., 2019), and resistance to chemical denaturation (Akram et al., 2018), and they could reduce contamination from unwanted microbes (Ebaid et al., 2019). Thermozymes also exhibit low fluid viscosity, and high saccharification and production yield (Sarmiento et al., 2015), thereby improving the cost-effectiveness of the bioprocess (Arora et al., 2015).

Thermophiles and hyperthermophiles are excellent sources of thermozymes for potential applications in industries and molecular biology. In particular, *Thermotoga maritima* (*Tma*), an anaerobic rod-shaped bacterium, with an optimal growth temperature of ∼80°C is considered an invaluable source of industrially important thermozymes (Myung et al., 2010). In fact, the genes TM1227 and 1751 from *Tma* encoded β-mannanases, have specific activities of up to 90 and 69 U/mg on galactomannan, respectively (Chhabra et al., 2002).

Considering the widespread usage of β-mannanases in different industries, there is a dire need to identify hyperthermophilic β-mannanases with high specific activity and thermostability. TM1752 (TM_RS08910) in the *Tma* genome encodes a putative endoglucanase Cel5B, belonging to the GH5 family. In this work, high level expression of gene TM1752 was attained in *Escherichia coli* using heat shock pHsh vector. Further, the recombinant protein was purified and biochemically characterized. To assess the possible application of the purified enzyme in the food/feed industry, the enzyme was used for the production of MOS from LBG which was evaluated by thin layer chromatography (TLC) analysis.

## Materials and Methods

### Chemicals, Strains, and Plasmid

Locust bean gum, galactomannan extracted from the seeds of a locust bean tree with mannose: galactose ratio of approximately 4:1, was purchased from Adamas (Pennsylvania Avenue, Washington, DC). Xylan, avicel, and carboxy methyl cellulose (CMC) were purchased from Merck (Singapore), and lichenan was purchased from Megazyme (Ireland). All other chemicals used in this study were of analytical grade and obtained from Sinopharm Chemical Reagent Co., Ltd. (China).

The *Tma* MSB8 (ATCC 43589) strain was acquired from the American Culture Collection (Manassas, Virginia), and grown in the *Thermotoga* basal medium (TMB) supplemented with 0.5% glucose (Jiang et al., 2006). Host *E. coli* JM109 was purchased from Promega Corporation (Madison, WI, United States), whereas the pHsh vector was provided by Shine E Biotech (Nanjing, China).

### Cloning of TM1752 Gene in Expression Plasmid pHsh

To amplify TM1752 gene (accession no. TM_RS08910), PCR was carried out using the genomic DNA of *Tma* MSB8 as a template. Primers were synthesized based on the open reading frame sequence with forward and reverse sequences TM1752F/TM1752R as follows: 5′-AATAACACCATTCCAAGATGG-3′/5′-GTATCTAGATTCA ATGCTATCTCCTCCTAA-3′. pHshF/pHshR: 5′-GTATCTAGA CACCACCACCACCACCACTAA-3′/5′*CAT*GGGTATATCTCC TTCTTGTC-3′. The primers were used to amplify the whole vector “pHsh” by reverse PCR (italic means the starting codon, and the underline indicates the *Xba*I recognition site). The amplified DNA fragments and the pHsh vector were digested using the restriction enzyme *Xba*I (Takara, the 5′ terminus is the blunt end and the 3′ teminus is the cohesive end), and purified by gel electrophoresis using Axygen AxyPrep DNA Gel Recovery Kit (Corning, NY, United States) according to the manufacturer’s instructions, and then ligated together using Takara Bio DNA Ligation Kit (Kyoto, Japan). The recombinant DNA was transformed into *E. coli* JM109, and the transformed *E. coli* cells were spread-plated onto Luria-Bertani (LB) solid agar plates supplemented with 100 μg/mL ampicillin. These plates were then kept at 30°C overnight. The positive transformants for the expression plasmid pHsh-TM1752 were selected for PCR and sequence verification.

### Gene Expression and Protein Purification

The recombinant plasmid was transformed into *E. coli* JM109 using the heat-shock method. Briefly, the transformed cells were grown on LB agar plates and incubated at 30°C for 24 h. One colony was transferred to 200 mL LB media supplemented with 100 μg/mL ampicillin and incubated at 30°C with shaking at 200 rpm up to an OD_600_ of 0.8. Subsequently, the cells were incubated at 42°C for 8 h for protein production.

Next, the cells were harvested by centrifugation at 7000 × *g* for 20 min at 4°C, the supernatant was discarded and the obtained cell pellets were resuspended in the binding buffer (5 mM imidazole, 0.5 M NaCl, and 20 mM Tris) at pH 8.0. Then, the cells were disrupted by sonication for 30 min (9 s on and 6 s off). The obtained cytoplasmic extract was subjected to heat treatment at 70°C for 20 min and centrifuged at 10, 000 × *g* for 30 min at 4°C to remove denatured protein and cell debris. The supernatant was loaded onto the immobilized nickel-affinity column (GE) equilibrated with the binding buffer, and eluted using the elution buffer (500 mM imidazole, 0.5 M NaCl, and 20 mM Tris–HCl) at pH 8, separated by sodium dodecyl sulfate-polyacrylamide gel electrophoresis (SDS–PAGE), and dialyzed against phthalate-imidazole (PI) buffer at pH 6.8 for subsequent analysis. The purified enzyme was conserved in the stock solution (25 mM pH 6.8 of PI buffer, 20% glycerol, and 0.02% sodium azide). Protein concentration of the purified enzyme was determined by measuring the absorbance at 280 nm (A280).

### Enzyme Assay and Substrate Specificity

The activity of the recombinant enzyme was determined using the 4-hydroxybenzoic acid hydrazide method (Lever, 1972). The reaction mixture contained 100 μL of 0.5% (*w/v*) substrate in water, 95 μL of PI buffer (25 mM, pH 6.0), and 5 μL of purified enzyme, was incubated at the target temperature for 5 min. The reaction was stopped by adding 600 μL of 4-hydroxybenzoic acid hydrazide and boiling for 10 min. The reaction mix was cooled on ice, and the absorbance was measured at 410 nm. One unit (U) of enzyme activity is defined as the quantity of enzyme required to release 1 μmol of reducing sugar in 1 min. All enzyme assays were performed in triplicate, and data were reported as the mean ± standard deviation.

To examine the specificity of the recombinant enzyme toward different substrates, the enzyme was incubated with LBG, CMC, avicel, xylan, lichenan, and starch (0.5%, *w/v*) at 85°C and pH 5.5. The reaction mixture also contained 1 mM CoCl_2_.

### Sequence Analysis

The amino acid sequence of TM1752 was compared with that of proteins in the gene bank via BLAST analysis on the NCBI server. Multiple sequence alignment was performed using Clustal W (1.83), as displayed by Escript. The molecular mass of recombinant enzyme was predicted using the Expasy bioinformatics resource portal.

### Biochemical Charaterization of Purified Enzyme

The optimum temperature of the purified enzyme was determined at various temperatures from 60 to 90°C using assay conditions as mentioned earlier in 25 mM PI buffer at pH 6.8. Thermostability was determined by incubating the enzyme at 85, 90, and 95°C without substrate in the PI buffer (pH 6.8) for up to 5 h. The incubated enzyme samples were taken after every hour, and residual activity was determined under standard assay conditions as described earlier.

The optimum pH of the recombinant enzyme was examined at pH ranging from pH 4.0 to 7.5, using 25 mM PI buffer at the optimal temperature. pH stability was evaluated by calculating the residual enzyme activity at optimum conditions after incubation in PI buffer of pH 3.0–8.0 at optimal temperature for 1 h without substrate.

The impact of detergents and oxidizing and reducing agents, including SDS, Tween-80, Triton X-100, CTAB, hydrogen peroxide, ammonium persulfate, Ascorbic acid, and EDTA (at a final concentration of 1%), on the enzyme activity of the recombinant enzyme was examined by pre-incubating the enzyme in the presence of these preparations for 30 min. The remaining enzymatic activities were estimated at optimum temperature and pH. The activities of the purified enzyme without any additives and pre-incubated at same conditions were recorded as 100% activity.

Furthermore, the influence of Cu^2+^, Ni^2+^, Co^2+^, Mn^2+^, Ca^2+^, Cr^3+^, Zn^2+^, Mg^2+^, and Sr^2+^ ions was examined by adding 1 mM of each ion to the reaction mixture. Then, enzyme activity was determined at the optimal temperature and pH, and recorded as the percent of enzyme activity observed when no metal ions were added to the reaction system. The metal with the most significant effect on enzyme activity was selected, and its optimum concentration required to increase enzyme activity under optimal reaction conditions was determined. To this end, the enzyme activity was determined at increasing concentration (0.0–2.0 mM) of selected metal ion. The enzyme activity was determined under optimal reaction conditions.

Kinetic parameters were calculated by observing enzyme activity in increasing substrate concentrations i.e., 0.5–15.0 mg/mL and 1 mM Co^2+^. The Michaelis–Menten constant (K_*m*_) and the maximum rate of reaction (V_*max*_) were determined using the Michaelis–Menten (V vs. [S]) and Lineweaver–Burk plots (1/V vs. 1/[S]).

### Hydrolysis Products Analysis

Thin layer chromatography was employed for the examination of sugar products liberated in the hydrolysis of LBG by recombinant enzyme. In this regard, 100 μL of LBG (0.5%) dissolved in PI buffer (pH 5.5) and 100 μL purified enzyme (0.364 mg/mL) were mixed together and incubated at optimum temperature for 15, 30, 45, and 60 min. Then, the mixture was boiled for 10 min, and centrifuged at 7000 × *g* for 5 min, and the supernatant was frozen till volume reached 10 μL.

The TLC plate (Silica gel-coated aluminum plate, Merck, Germany) was spotted with 1.0 μL of concentrated hydrolyzed sample and standards of mannose, mannobiose and mannotriose. The concentration of the standards was 1 mg/mL in ddH_2_O. The plate was developed using acetonitrile: water (65:35 *v/v*) as the mobile phase. After drying, the plate was stained with 0.3% N-(1-napthyl)ethylenediamine dihydrochloride dissolved in methanol containing 5% H_2_SO_4_ (Bounias, 1980) to detect the sugars.

## Results

### Gene Expression and Enzyme Purification

**FIGURE 1:**
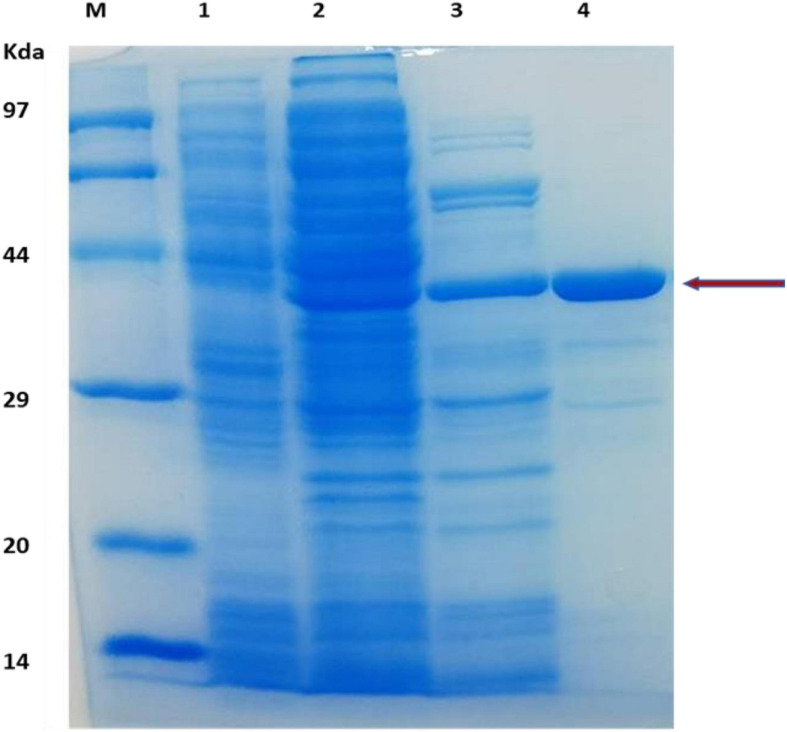
SDS-PAGE analysis of protein obtained during gene expression and protein purification. Lanes: M, protein makers for molecular mass; 1, *E. coli* cell protein as a control; 2, Cell-free extract of *E. coli* expressing TM1752; 3, After heat treatment of cell-free extract; 4, Purification of the recombinant protein.

The TM1752 gene was successfully amplified from genomic DNA of *Tma* via PCR, ligated with vector pHsh and transformed into *E. coli* using heat shock method. The gene was expressed to a relatively high level in pHsh expression system (Figure 1). The recombinant enzyme was purified via subsequent heat treatment and Nickle affinity chromatography of the soluble fraction of cells obtained after sonication. The enzyme was purified to near homogeneity as depicted in SDS-PAGE analysis (Figure 1). The purified enzyme exhibited a molecular mass of approximately 38 kDa in SDS-PAGE, which was in agreement with the prophesied (ExPasy online portal) molecular mass of 39 kDa.

### Substrate Specificity of Recombinant Enzyme

The specificity of the purified enzyme with different carbon sources was examined by incubating the enzyme with LBG, CMC, avicel, lichenan, starch, and xylan. These substrates vary in their backbone composition and the type of glycosidic linkages. The purified protein exhibited the highest activity of 416 U/mg for LBG (Table 1), a galactomannan. Among glucan-based substrates, the strongest enzyme activity for CMC (215 U/mg) and a very low activity of 1.42 for Avicel were observed. The specific activity of the purified enzyme for lichenan was only 0.88 U/mg. The enzyme however didn’t show any activity for starch and xylan.

**TABLE 1:** Substrate specificity of the purified protein from expression plasmid pHsh-TM1752.

Substrate	Backbones (type of bond or linkage)	Specific activity (U/mg)
Locust bean gum	β-1,4-glycosidic bond (mannose)	416
Carboxy methyl cellulose	β-1,4-glycosidic bond (glucose)	215
Avicel	β-1,4-glycosidic bond (glucose)	1.42
Starch	α-1,4-glycosidic bond (glucose)	0
Xylan	β-1,4-glycosidic bond (xylose)	0
Lichenan	β-1,3-1,4- glycosidic bond (glucose)	0.88

### Bioinformatic Analysis of TM1752 Gene Product

**FIGURE 2:**
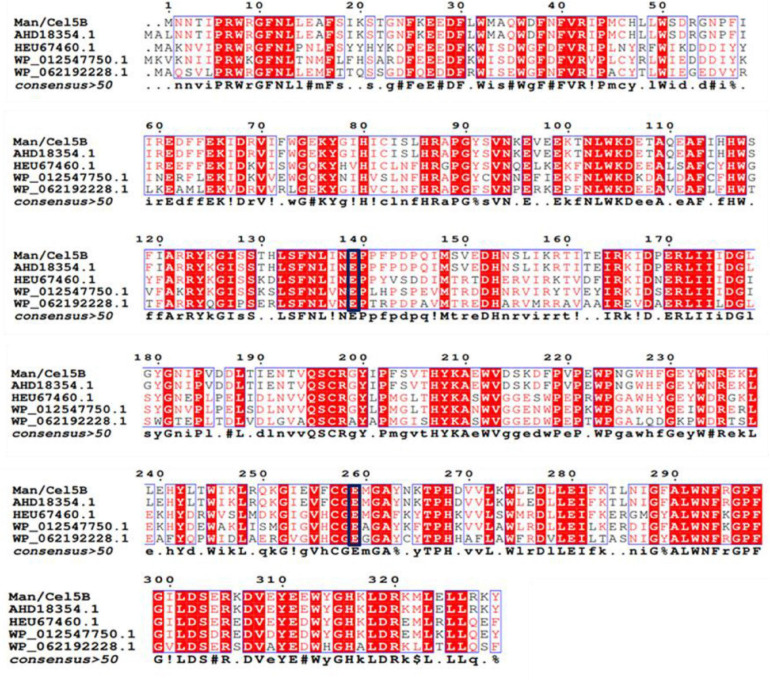
Comparison of amino acid sequence of TM1752 gene with enzymes deposited in GenBank database. The enzymes include endoglucanase from *Thermotoga maritima* (accession number AHD18354.1), *Dictyoglomus thermophilum* (accession number HEU67460.1), *Dictyoglomus thermophilum* (accession number WP_012547750.1), and *Anaerolinea thermolimosa* (accession number WP_062192228.1). The amino acids in the black box represent putative catalytic amino acid residues.

The deduced amino acid sequence of TM1752 gene was paralleled with protein sequenes of already-characterized proteins using BLAST. According to the amino acid homology search (Figure 2), Cel5B did not show similarity with any other β-mannanase in the GenBank. It demonstrated highest identity (99.7%) with endoglucanase from *Tma* (accession number AHD18354.1). The amino acid sequence homology with other endoglucanases were 68.6, 58.6, and 53.9% from *Dictyoglomus thermophilum* (accession number HEU67460.1), *D. thermophilum* (accession number WP_012547750.1), and *Anaerolinea thermolimosa* (accession number WP_062192228.1), respectively. These results indicate that Cel5B is principally a novel mannanase as it exhibited high specific activity for LBG and did not show sequence identity with already reported mannanases. Meanwhile, it had a sequence of up to 68.6% homology with the cellulases from some other thermophilic species other than *Tma*, and showed considerable specific activity for CMC as well. Therefore, the enzyme is a novel bifunctional mannanase/cellulase that should be renamed as Man/Cel5B. Besides, the amino acid sequence analysis predicted that Glu 139 and Glu 259 worked as acid/base and nucleophile residues, respectively during hydrolysis of substrate by Man/Cel5B.

### Catalytic and Kinetic Properties of Man/Cel5B

**FIGURE 3:**
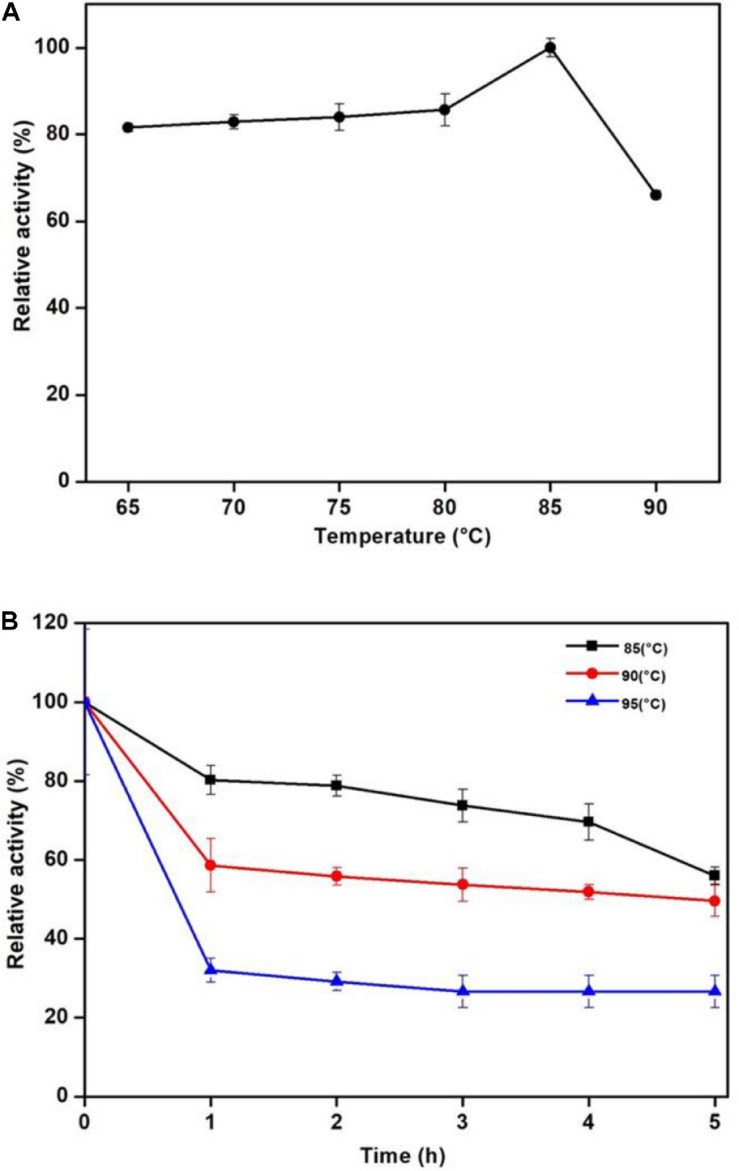
Effect of temperature on the mannanase activity of Man/Cel5B. **(A)** Dependence of mannanase activity on the temperature. **(B)** Thermostability of Man/Cel5B. The enzyme (0.364 mg/mL) was incubated at temparatures 80, 85, and 90°C for 5 h in the absence of substrate, and residual activity was examined at optimal conditions.

**FIGURE 4:**
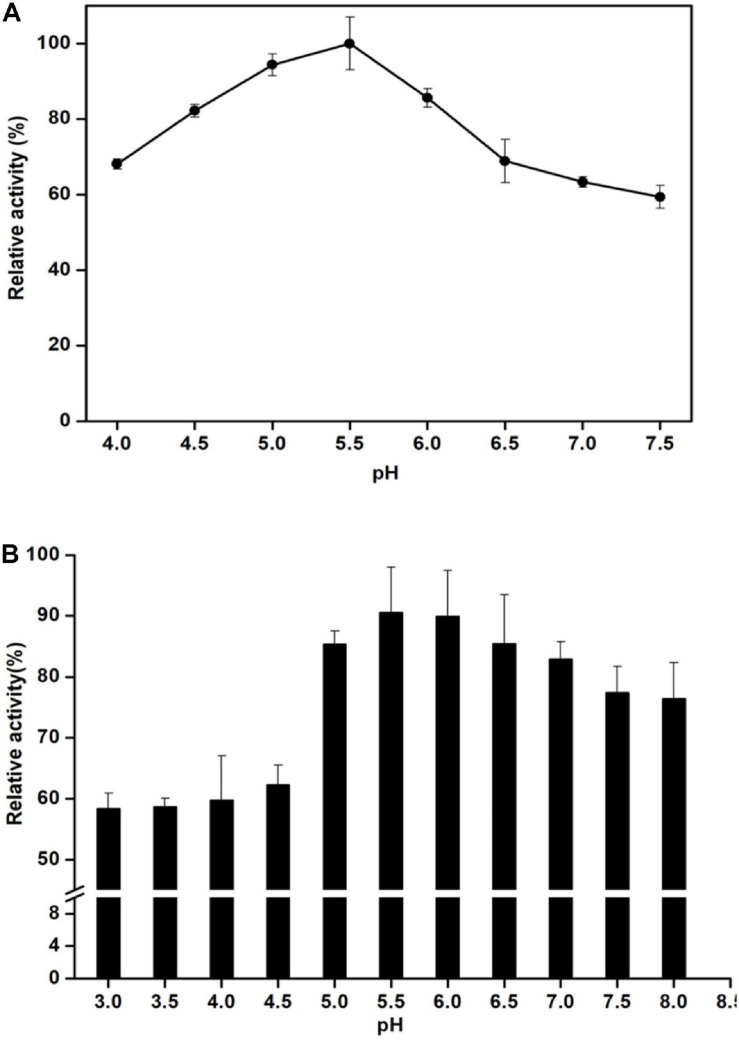
Effect of pH on the mannanase activity of Man/Cel5B. **(A)** Determination of pH optimum. Enzyme activity was determined in Phthalate-Imidazole (PI) buffer with pH ranging between 4 and 8. **(B)** Enzyme stability at various pH. The enzyme was incubated in PI buffer with different pH values at a range of 4–7.5 for 1 h in the absence of substrate, and residual activity was measured by adding substrate dissolved in the buffer at pH 5.5.

Man/Cel5B exhibited enzyme activity over the range of studied temperature. Its activity reached the highest at 85°C and drastically reduced at 90°C (Figure 3A). Thermostability experiments specified that Man/Cel5B retained >55% of the activity after 5 h of pre-incubation in the absence of substrate at 85°C and >50% of the enzyme activity after 4 h at 90°C (pH 6.8) (Figure 3B). The purified enzyme exhibited more than 70% of activity between pH 5.0 and pH 6.0 with an optimum pH of 5.5 (Figure 4A). In addition, after pre-incubation for 1 h in PI buffer having pH values between 3.0 and 8.0 (85°C), Man/Cel5B retained >85% of the initial activity at pH range 5.0–7.0 (Figure 4B). The highest stability of Man/Cel5B was observed at pH 6.0 where the enzyme retained 96% of its initial activity.

The influence of various chemicals on the activity of Man/Cel5B are summarized in Table 2. Among the tested chemicals, Tween 80 enhanced enzyme activity by up to 137%. Hydrogen peroxide, ascorbic acid and ammonium persulfate similarly boosted its activity to 112, 109, and 102%, respectively (Table 2). On the other hand, EDTA slightly reduced enzyme activity to 94%. Furthermore, only SDS and CTAB inhibited the enzyme activity to a greater extent, that is 49 and 53%, respectively.

**TABLE 2:** Effect of detergents, oxidizing agents, reducing agents, and chelating agents on the Man/Cel5B activity.

Reagents	Concentration	Relative activity (%)
Control		100%
Detergents	1% (*w/v*)	
SDS	1% (*w/v*)	43
CTAB	1% (*w/v*)	59
Tween-80	1% (*v/v*)	137
Triton X 100	1% (*v/v*)	74
**Oxidizing agents**		
Ammonium persulfate	1% (*w/v*)	103
Hydrogen peroxide	1% (*w/v*)	113
Reducing agent		
Ascorbic acid	1% (*v/v*)	110
Chelating agent		
EDTA	1% (*w/v*)	94

**FIGURE 5:**
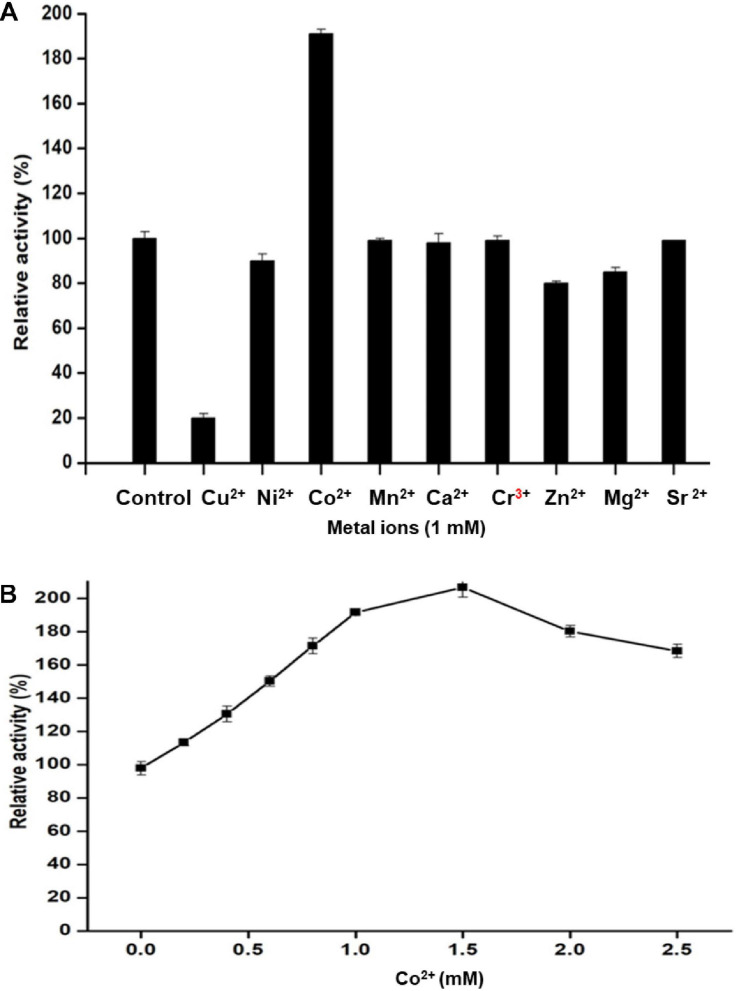
Effect of different metal ions on Man/Cel5B activity. **(A)** Mannanase activity in the presence of a metal ion at final cocentration of 1 mM. **(B)** Effect of Co^2+^ ion concentration on the enzyme activity. Co^2+^ was added to the reaction system in increasing concentration (0.0–2.5 mM), and relative activity was evaluated at optimal reaction conditions.

The influence of ionic metals on Man/Cel5B activity was examined by determining the mannanase activity in the presence of 1 mM metal ion in the reaction mixture (Figure 5A). According to the differential analysis, Co^2+^ significantly enhanced the enzyme activity to 191% as compared to control (activity when no ion was added). However, the enzyme activity was drastically reduced to 20% of its initial activity in the presence of Cu^2+^, thereby indicating its inhibitory effect on Man/Cel5B. In addition, metals like Ni^2+^ and Zn^2+^ exhibited slight inhibition of enzyme activity by about 15%. The metal ion requirement for achieving high activity was further examined in the presence of increasing Co^2+^ concentrations. The optimum concentration of Co^2+^ for Man/Cel5B activity was between 1.0 and 1.5 mM (Figure 5B), as the enzyme activity increased to approximately 200%, whereas the enhancement of Man/Cel5B activity started reducing when Co^2+^ concentration reached 2 mM or higher. The K_*m*_ and V_*max*_ values were evaluated at optimum conditions of temperature and pH with 1 mM Co^2+^ in the increasing concentration of LBG. K_*m*_ and V_*max*_ were found to be 4.5 mg/mL and 769 U/mg, respectively.

### Analysis of LBG Hydrolysis Products

**FIGURE 6:**
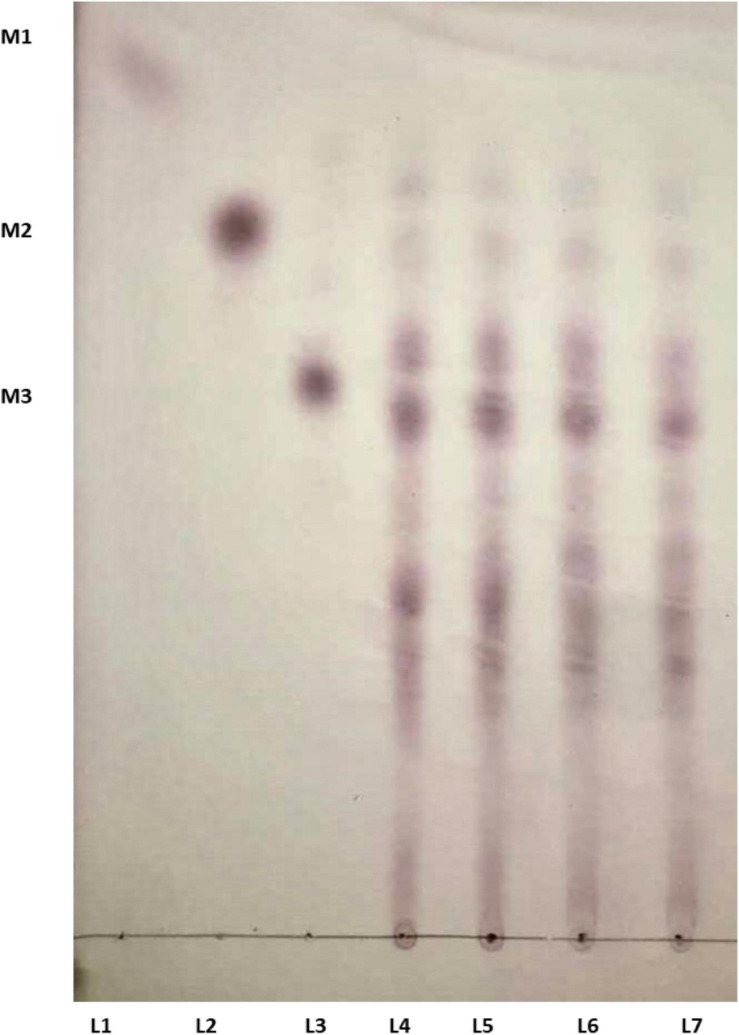
TLC analysis of the hydrolysis products of LBG by Man/Cel5B. Equal volume of enzyme and LBG were incubated together for 15 min (L 4), 30 min (L 5), 45 min (L 6), and 60 min (L 7). Lanes 1–3 represents standards mannose (M1), mannobiose (M2), and mannotriose (M3).

The time course enzymatic hydrolysis products of LBG were analyzed, and the produced mannobiose, mannotriose, and MOS were detected on TLC plates (Figure 6). LBG was not hydrolyzed into mannose, which indicates Man/Cel5B cleaves internal bonds in the mannose back bone. The production of mannobiose, mannotriose, and MOS upon degradation of LBG indicates that Man/Cel5B can be used for the hydrolysis of commercially available LBG for the production of MOS as well as the hydrolysis of mannan in plant materials such as soybean meal or coffee bean powder.

## Discussion

Among all the thermophilic sequences currently available, the genomic sequence of *Tma* encodes highest (7%) number of glycosyl hydrolases. Nevertheless, many of these putative glycosyl hydrolases have not been further characterized. TM1752 gene from *Tma* encodes putative endoglucanase named as Cel5B. In this study, we report that TM1752 encodes for a novel bifunctional enzyme which can degrade mannan and cellulose with the highest activity for mannan. In substrate specificity assay, many enzymes are found to have a low activity on some other substrates with structures close to their preferred substrates. However, in the work reported here, the enzyme produced from the cellulase gene TM1752 has been found to use natural galactomannan as a main substrate, and therefore to have a mannanase activity much higher than its cellulase activity. These findings not only reveal a hyperthermophilic enzyme with the highest mannanase activity (Table 3), but also offer a bifunctional enzyme that is highly active to both mannan and cellulose (Table 1), which is potentially applied to an effective degradation of lignocellulosic biomass.

**TABLE 3:** A comparison of properties of Man/Cel5B with some previously reported thermophilic and hyperthermophilic GH 5 β-mannanases.

Organism/gene	Temperature optimum (°C)	pH optimum	Temperature and pH stability	K_*m*_ value mg/ml	Specific activity (U/mg)	Molecular mass (kDa)	References
*Thermotoga maritima^a^* TM1752	85	5.5	5 h 85°C, 5–8	4.5	416	38	This study
*T. neapolitana* TN5068	91	7.1	NG	NG	1.23	55	McCutchen et al., 1996
*T. maritima^a^* TM1227	90	7	NG	NG	72	76.9	Chhabra et al., 2002
*Dictyoglomus turgidum^a^*	70	5.4	2 h 70°C, 5–9	4.70	216.96	40	Fusco et al., 2018
*Phialophora sp. P13^a^*	60	1.6	2 h 55°C, 1.5–7	2.5	851	44.2	Zhao et al., 2010
*Bacillus* sp. N16-5	70	9.5	2 h 60°C, 8.5–10	NG	NG	50	Zheng et al., 2016
*B. subtilis* YH12	55	6.5	NG	30	7302.4	40	Liu et al., 2015
*Bacillus circulans* CGMCC 1416^a^	58	7.6	1 h 50°C, 7–9	NG	NG	31	Yanan et al., 2008

*^a^Means recombinant enzyme.*

*NG means not given.*

Hyperthermophilic enzymes have been found to be produced at very low levels, or to form inclusion bodies at large extent, only a small proportion of these enzymes are soluble and active (Yu et al., 2016). In the present study, pHsh vector was used for the expression of TM1752 gene in *E. coli*. pHsh utilizes a heat shock (Hsh) promoter for controlling the inserted gene, where alternative σ^32^ regulates the gene expression. This plasmid is thought to have a high copy number of approximately 200 in host *E. coli*, and an upsurge in the temperature causes a swift increase of σ^32^ which causes immediate expression of the target gene (TM 1752 in this case). A large number of chaperon proteins are also synthesized in *E. coli*, which increase the chance for the soluble expression of protein (Sha et al., 2020).

The substrate specificity of the purified enzyme depicts its dual nature as the enzyme could degrade both LBG and CMC. However, the mannanase activity was two-fold higher than its cellulase activity on CMC, and four-fold higher than the highest activity reported earlier for mannanase from *Tma* on galactomannan (Chhabra et al., 2002). The paired activity for both glucan- and mannan-based polysaccharides is a rare phenomenon for enzymes belonging to GH5 family, however there are few reports where β-mannanase has been found to degrade cellulose as well such as, Cel5A encoded by TM1751 gene of *Tma* (Chhabra et al., 2002), DturCelA and DturCelB of *Dictyoglomus turgidum* (Brumm et al., 2011; Fusco et al., 2018). According to Pereira et al. (2010), the structural analysis of cellulases and mannanases belonging to GH5 family revealed similarity in the active sites of both enzymes, which could be responsible for bifunctional nature of these enzymes. The structural analysis of GH5 cellulases and GH5 mannanases have revealed that the catalytic clefts of these enzymes use same active sites for hydrolysis of cellulose and mannan, respectively. The active sites of these enzymes contain two conserved glutamate residues that act as acid/base and nucleophile during hydrolysis of the substrate (Sakon et al., 1996; Hilge et al., 1998). The 3D structure of Man/Cel5B has been resolved, and deposited in protein data base bank (PDB code 1vjz) (Data not published), which revealed that Glu 139 and Glu 259 are used as active site residues for the hydrolysis of substrate by this enzyme. The similarity in the active sites of GH5 cellulases and GH5 mannanases could be a possible reason for the dual nature of Man/Cel5B. However, to gain the in-depth knowledge of dual substrate recognition mechanism, the cellulose and mannan bound crystal structure of Man/Cel5B should be compared with apoenzyme in future. This will be helpful in the functional improvement of Man/Cel5B and other enzymes by protein engineering. The excellence to degrade both mannan and cellulose may play a significant role in the complete degradation of complex lignocellulosic material for subsequent biofuel production. Furthermore, the specific activity of most of the earlier-reported hyperthermophilic GH5 mannanases was much lower than that of β-mannanase reported in the current study (Table 3). β-mannanases from *Phialophora sp. P13* (Zhao et al., 2010) and *Bacillus subtilis YH12* (Liu et al., 2015) have higher specific activity of 851 and 7302 U/mg, respectively for LBG, however, the optimum temperature of these enzymes is quite low compared with that of Man/Cel5B. The high specific activity and high thermotolerance of Man/Cel5B makes it an excellent candidate for industrial applications.

The optimum temperature of Man/Cel5B (85°C) is higher than those of most β-mannanases that typically have optimum temperature between 45 and 75°C (Luo et al., 2017). Though, the β-mannanase from *Tma* (TM1227) (Chhabra et al., 2002) and *Thermotoga neapolitana* (McCutchen et al., 1996) have slightly higher optimal temperatures of 90 and 91°C, respectively. However, the specific activity of these enzymes is very low (90 and 55 U/mg, respectively) compared with that of Man/Cel5B (416 U/mg). A sudden decrease in enzyme activity was observed at 90°C which is thought to be because of denaturation of the protein at higher temperature (Dar et al., 2019). One of the important characteristics of Man/Cel5B is its longstanding thermostability which is higher than most of thermophilic and hyperthermophilic β-mannanases belonging of GH5. Thermostability was only comparable to *Tma* (TM1227) which lost almost 50% of the initial activity after 3 h of incubation at 90°C (Chhabra et al., 2002). β-mannanase from *T. neapolitana* has been reported to retain 50% of the initial activity at 91°C after 13 h of incubation (McCutchen et al., 1996). However, the gene encoding this protein has not been annotated to date. The optimum pH for Man/Cel5B was found to be in acidic range which is similar to that for β-mannanase (*Dtur*CelB) from *D. turgidum* (Fusco et al., 2018). However, the optimum pH for β-mannanase from *Tma* TM1227 is pH 7.1 (Chhabra et al., 2002), which shows the optimum pH of β-mannanase is not species-specific.

The activity of Man/Cel5B was stable in the presence of most of the tested chemicals and detergents. Interestingly, Man/Cel5B activity dramatically increased in the presence of Tween 80, and this may be attributed to increased substrate stabilization and decreased enzyme immobilization (Kamande et al., 2000). The presence of EDTA had almost no effect which indicates that Man/Cel5B is not metalloenzyme. Similarly, EDTA displayed no effect on β-mannanase activity from *Streptomyces* sp. S27 (Shi et al., 2011) and for *Dtur*CelB (Fusco et al., 2018). However, there are many reports where β-mannanase activity was either reduced to greater extent or completely halted in the presence of EDTA (Liu et al., 2015; Regmi et al., 2016; Singh et al., 2019). In this study, only SDS and CTAB drastically inhibited enzyme activity. Because SDS and CTAB can alter the three-dimensional structure of enzymes and consequently inhibit their activity (Singh et al., 2019). Similar effects of these ionic detergents were also observed on *Dtur*CelB (Fusco et al., 2018). Nevertheless, the activities of β-mannanase from *Bacillus* sp. N16-5 (Zheng et al., 2016), and *Bacillus* sp. CSB39 (Regmi et al., 2016) were enhanced in the presence of SDS.

In agreement with the present results, β-mannanase from *Phialophora* sp. P13 (MAN5AP13) and *Bacillus nealsonii* PN-11 also exhibited enhanced activity in the presence of Co^2+^ (Zhao et al., 2010; Chauhan et al., 2014). According to Andreini et al. (2008), metal ions enhance enzyme activity by assisting in the proper substrate binding at the active sites. The activity of Man/Cel5B started decreasing when the concentration of Co^2+^ exceeded 1.5 mM in the reaction mixture. The decrease in the activity at higher concentration of Co^2+^ ion may be due to the toxicity effect on enzyme (Afkar et al., 2010). Among the tested chemicals, only Cu^2+^ decreased the enzyme activity dramatically. Our results are supported by the observations of Fusco et al. (2018), who reported that the activity of *Dtur*CelB was greatly reduced in the presence of Cu^2+^. Similar inferences have been proposed for MAN5AP13 (Zhao et al., 2010). The change in enzyme activity in the presence of ions may be due to an alteration in the net overall electric charge of the enzyme surface (Zheng et al., 2016). The V_*max*_ for Man/Cel5B was much higher than the already reported thermostable β-mannanases, for example, V_*max*_ for β-mannanase from *D. turgidum* (Fusco et al., 2018) and *Thermotoga petrophila* (Santos et al., 2012) were 473.1 and 114 U/mg, respectively.

The substrate binding mechanism and the end products produced by the degradation of mannan containing polysaccharides by β-mannanases vary to a large extent even within the same family (Katrolia et al., 2013). The extent of the substitution of galactose residues and the distribution of glucose residues on the main chain of the mannan backbone affects the pattern of hydrolysis of mannan polysaccharides (Katrolia et al., 2013). The majority of β-mannanases cleave the mannan polysaccharide into mannobiose and mannotriose, yet there are few reports where mannose is also produced. Man/Cel5B hydrolyzes LBG into mannobiose, mannotriose and MOS. This hydrolysis pattern of Man/Cel5B is also shared by mannanase from *Cellulosimicrobium* sp. HY-13 (Kim et al., 2011). However, those from *Bacillus* sp. N16-5 (Ma et al., 2004), and *Streptomyces* sp. CS428 (Pradeep et al., 2016) resulted in the formation of mannose and MOS. MOS have gained a lot of interest as prebiotics. Because MOS cannot be digested by gastric or pancreatic juice and are utilized by the gut microbial flora for the synthesis of short-chain fatty acids. These fatty acids promote the growth of beneficial gut flora and are also used as energy source by the host organism (Asano et al., 2003). In fact, gammanase and hemicel-mannanase (commercially available β-mannanase) are currently utilized as prebiotics in poultry feed to mitigate the immunogenic effect of mannan (Korver, 2006). Srivastava and Kapoor (2017) used MOS produced by hydrolysis of LBG from *Streptomyces* sp. CS428 to improve the growth of various *Lactobacillus* species (beneficial gut bacteria) and also food born pathogenic bacteria such as *E. coli* and *Salmonella typhi*. These results proposed that MOS enhances the growth of *Lactobacillus* species, whereas inhibited the growth of *E. coli* and *S. typhi*. β-mannanase can also be used in the preparation of instant coffee to degrade galactomannan (results in gel formation during freeze-drying) in liquid coffee. All these observations suggest that Man/Cel5B has a wide potential for application in the food and feed industries as prebiotic for the production of MOS.

## Conclusion

In conclusion, TM1752 from *Tma* was cloned and overexpressed in *E. coli* and indentified to be bifunctional mannanase and cellulase (Man/Cel5B). The purified Man/Cel5B enzyme was optimally active at 85°C and pH 5.5, and exhibited high specific activity 416 U/mg for LBG, which is the highest among those of the hyperthermophilic mannanases reported earlier. In addition, Man/Cel5B displayed high thermal and pH stability, and could efficiently degrade LBG into mannobiose, mannotriose and MOS. All these properties make Man/Cel5B an excellent candidate for future food/feed and biofuel industries.

## Data Availability Statement

The original contributions presented in the study are included in the article/supplementary material, further inquiries can be directed to the corresponding author/s.

## Author Contributions

WS and CS comprehended and designed the experiments. BS and WZ performed the experiments. BS and PR analyzed the data. BS, CS, HW, and WS wrote and revised the manuscript. All authors contributed to the article and approved the submitted version.

## Conflict of Interest

The authors declare that the research was conducted in the absence of any commercial or financial relationships that could be construed as a potential conflict of interest.
